# *Bacillus telluris* sp. nov. Isolated from Greenhouse Soil in Beijing, China

**DOI:** 10.3390/microorganisms8050702

**Published:** 2020-05-10

**Authors:** He-Bao Guo, Shan-Wen He, Xing Wang, Kyu-Kyu Thin, Hai-Lei Wei, Xiao-Xia Zhang

**Affiliations:** Key Laboratory of Microbial Resources Collection and Preservation, Ministry of Agriculture and Rural Affairs, Institute of Agricultural Resources and Regional Planning, Chinese Academy of Agricultural Sciences, Beijing 100081, China; guohebao0409@163.com (H.-B.G.); 201772472@yangtzeu.edu.cn (S.-W.H.); 82101182006@caas.cn (X.W.); 2018y90100142@caas.cn (K.-K.H.)

**Keywords:** *Bacillus telluris* sp. nov., genome analysis, plant-growth promoting rhizobacterium

## Abstract

A novel Gram-stain-positive, rod-shaped, endospore-forming bacterium, which we designated as strain 03113^T^, was isolated from greenhouse soil in Beijing, China. Phylogenetic analysis based on 16S rRNA gene sequences showed strain 03113^T^ is in the genus *Bacillus* and had the highest similarity to *Bacillus solani* CCTCC AB 2014277^T^ (98.14%). The strain grew at 4 °C–50 °C (optimum 37 °C), with 0–10% (*w/v*) NaCl (optimum 5%), and in the range of pH 3.0–12.0 (optimum pH 8.0). Menaquinone was identified as MK-7, and the major polar lipids were diphosphatidylglycerol, phosphatidylglycerol, and phosphatidylethanolamine. The main major cellular fatty acids detected were anteiso-C_15:0_ (51.35%) and iso-C_15:0_ (11.06%), which are the predominant cellular fatty acids found in all recognized members of the genus *Bacillus*. The 16S rRNA gene sequence and core-genome analysis, the average nucleotide identity (ANI), and in silico DNA—DNA hybridization (DDH) value between strain 03113^T^ and the most closely related species were 70.5% and 22.6%, respectively, which supported our conclusion that 03113^T^ represented a novel species in the genus *Bacillus*. We demonstrated that type strain 03113^T^ (=ACCC 03113^T^=JCM 33017^T^) was a novel species in the genus *Bacillus*, and the name *Bacillus telluris* sp. nov. was proposed. Strain 03113^T^ secreted auxin IAA and carried the nitrogenase iron protein (*nifH*) gene, which indicated that strain 03113^T^ has the potential to fix nitrogen and promote plant growth. *Bacillus telluris* sp. nov. 03113^T^ is a potential candidate for the biofertilizers of organic agriculture areas.

## 1. Introduction

The genus *Bacillus* was first described by Cohn in 1872, and it is a genus of ubiquitous soil microorganisms [1]. It is comprised of endospore-forming, rod-shaped bacteria that are members of the phylum *Firmicutes* [2]. At the time of writing, there were 379 species in the genus *Bacillus* recorded on LPSN (www.bacterio.net/bacillus.html; Nov 2019). Based on their genetic similarity, *Bacillus* species can be classified into several groups, which include *Bacillus cereus*–*Bacillus anthraci*s–*Bacillus thuringiensis*, *Bacillus clausii*–*Bacillus halodurans, Bacillus coahuilensis*–*Bacillus* sp. NRRLB-14911, and *Bacillus subtilis*–*Bacillus licheniformis*–*Bacillus pumilus* [3]. In addition, species in the genus *Bacillus* have a wide range of physiological and biochemical characteristics from psychrophilic to thermophilic, acidophilic to alkaliphilic, and some are halophilic [4], which allow them to live in a wide range of extreme habitats, such as desert sands, hot springs, and Arctic soils. In addition, the genus *Bacillus* is an extremely diverse group of bacteria that includes both the causative agent of anthrax (*B. anthracis*) [5,6] and several species that synthesize important antibiotics. In addition to medical uses, bacillus spores, due to their extreme tolerance of both heat and disinfectants, are used to test heat sterilization techniques and chemical disinfectants. *Bacilli* are also used in the detergent manufacturing industry for their ability to synthesize important enzymes. 

In this study, we report a novel bacterial strain, 03113^T^, which was isolated from the greenhouse soil of Wangsiying, Chaoyang District, Beijing, China. Based on the phenotypic characteristics and phylogenetic analysis, strain 03113^T^ represents a novel species in the genus *Bacillus*.

## 2. Materials and Methods

### 2.1. Bacterial Strains, Growth Conditions, and Cultivation

Strain 03113^T^ was isolated from greenhouse soil from Wangsiying, Chaoyang District, Beijing, China (40°09′N, 116°42′E). We preserved the sample in freeze-dried milk ampoules at 4 °C and 20% (*v/v*) glycerol at −80 °C [7]. The type strains of species closely related to strain 03113^T^ were used as reference strains under the same conditions for comparative taxonomic analysis, which included *B. solani* CCTCC AB 2014277^T^, *B. praedii* CCTCC AB 2015208^T^, and *B. dabaoshanensis* CCTCC AB 2013260^T^_._ All strains were maintained and cultivated in TSA or TSB (Difco^TM^) medium plates at 30 °C, unless otherwise stated.

### 2.2. Phenotypic Characterization

Biochemical characteristics of strain 03113^T^ were investigated. Growth at eight different temperatures (4, 15, 25, 30, 37, 40, 45, and 50 °C) was tested on TSA plates. The pH values (pH 3.0, 4.0, 5.0, 6.0, 7.0, 8.0, 9.0, 10.0, 11.0, and 12.0, with increments of 1.0 pH unit) were tested in LB medium. Growth at various NaCl concentrations was tested over the range 0%−12% (*w/v*) NaCl (at intervals of 1%) by incubating at 30 °C [8]. Gram staining was performed using the Gram-stain kit [9]. Cell morphology was observed by light microscopy (CX21; Olympus) and transmission electron microscopy. Endospores were examined according to the Schaeffer–Fulton staining method [10]. Motility was examined on motility agar [11]. Catalase activity was determined by investigating bubble production with 3% (*v/v*) H_2_O_2_, and oxidase activity was determined using 1% (*v/v*) tetramethyl-*p*-phenylenediamine. The basic biochemical characteristics were investigated on API-20NE, API 50CH (BioMérieux) [12], and BIOLOG GEN III MicroPlate (BIOLOG), according to the manufacturer’s instructions. The type strains of *B. solani* CCTCC AB 2014277^T^, *B. praedii* CCTCC AB 2015208^T^, and *B. dabaoshanensis* CCTCC AB 2013260^T^ were used as reference strains.

### 2.3. Chemotaxonomic Analysis

For the measurement of chemotaxonomic characteristics, the menaquinone system was analyzed as described by Collins et al. [13] using reversed-phase HPLC [14]. The analysis of polar lipids by two-dimensional TLC was performed according to the method described by Minnikin et al. [15]. The cellular fatty acid is a useful and functional tool to identify species in the genus *Bacillus* and related genera. After 48 h of incubation at 30 °C on TSA, cellular fatty acids were extracted and analyzed using the method described by Sasser [16] and identified with the MIDI Sherlock Microbial Identification System (Library RTSA6 6.0, MIDI Sherlock Software Package, Version 6.0; Agilent 6890N). 

### 2.4. Phylogenetic 16S rRNA Gene Analysis

Genomic DNA was extracted from a single colony of the novel strain grown on TSA plates at 30 °C for 2 d using Bacteria DNA Kit (Tiangen, Beijing, China), according to the manufacturer’s protocol. The 16S rRNA gene was amplified by PCR and sequenced using the universal primers 27F(5’-AGAGTTTGATCCTGGCTCAG-3’) and 1492R(5’-GGTTACCTTGTTACGACTT-3’) [17]. Pairwise 16S rRNA gene sequence similarities were calculated using the EzTaxon-e database (http://eztaxon-e.ezbiocloud.net/) [18]. The CLUSTAL_W algorithm was used for sequence alignments using the neighbour-joining [19,20] and maximum-likelihood [21] methods that were implemented with Mega 7.0 software for phylogenetic analysis. Evolutionary distances were computed by using the Kimura two-parameter model [22]. The robustness of the tree branches was estimated by bootstrap analysis with 1000 replications [23]. The GenBank/EMBL/DDBJ accession number of 16S rRNA sequence is MN907472.

### 2.5. Complete Genome Sequencing and Analysis

To confirm the results of the 16S rRNA gene sequence similarity analysis, the complete genome sequence of the novel species was performed. The genome was sequenced by Personal Biotechnology Co., Ltd (Shanghai, PR China). Genomes of the most closely related species chosen above were retrieved from the GenBank database in NCBI. Reads of each data set were filtered by using AdapterRemoval (ver. 2.1.7) [24], and high-quality paired-end reads were assembled using A5-MiSeq v20150522 [25]. The open reading frames (ORFs) were predicted by GeneMarkS (ver. 4.32 April 2015) [26]. The tRNA genes were predicted by tRNAscan-SE 94 (ver. 1.3.1) and the rRNA genes by Barrnap (0.9-dev) 95 (https://github.com/tseemann/barrnap) [27]. Calculations of average nucleotide identity (ANI) were performed using JSpecies software (http://www.imedea.uib.es/jspecies). In silico DNA—DNA hybridization (DDH) estimates were performed using Genome-to-Genome Distance Calculator (GGDC) with the BLAST+ (recommended) method [28]. The partial genome files were uploaded to the GGDC 2.0 web interface (http://ggdc.dsmz.de/ggdc.php#), and Formula 2 was used as recommended for the calculation of DDH values. As a further extension of genome-based phylogeny, the GGDC website was used to establish the phylogenomic tree of strain 03113^T^ and other closely related *Bacillus* species. 

### 2.6. Analysis of Core Orthologous Genes

To identify orthologous genes among the strains in *Bacillus* species, 13 *Bacillus* strains were selected for the core genome analysis based on their biological control properties. The 13 bacteria included *B. solani* CCTCC AB 2014277^T^, *B. praedii* CCTCC AB 2015208^T^, *B. glycinifermentans* GO-13, *B. acidicola* FJAT-2406, *B. salacetis* SKP7-4, *B. shackletonii* LMG 18435, *B. circulans* NBRC 13626, *B. foraminis* Bac44, *B. persicus* DSM 25386, *B. oceanisediminis* CGMCC 1.10115, *B. firmus NCTC* 10335, *B. gottheilii* FJAT-2394, and 03113^T^. The Bacterial Pan Genome Analysis (BPGA) pipeline [29] was used for the pan-genome analyses. The clustering tool USEARCH was used to cluster protein families. The OrthoFinder [30,31] was used to perform an all-versus-all BLAST search based on nucleotide gene sequences of strain 03113^T^ and other related strains of the genus *Bacillus* to identify clusters of orthologous genes (OGs). Those OGs shared among all taxa and present in a single copy per genome were selected. They were aligned with Mafft [32] and subsequently concatenated. A phylogenetic tree based on orthologous proteins of the *Bacillus* genus was constructed by RA×ML version 8.2.12, based on the maximum-likelihood method. 

### 2.7. Plant Growth-Promoting Characteristics

The performance of secreting plant growth hormone indoleacetic acid (IAA) of strain 03113^T^ was measured by the PC Salkowski colorimetric method described by Glickmann and Dessaux [33]. The qualitative and quantitative analyses of siderophore production were conducted by the method described by Machuca and Milagres [34]. Phosphate solubilization was measured on inorganic and organic phosphate media [35]. All experimental analyses were performed in triplicate to ensure reproducibility. The results were expressed as the mean value of these determinations. 

## 3. Results and Discussion

### 3.1. Phenotypic Characterization of 03113^T^

The colonies of strain 03113^T^ were Gram-stain-positive and rod-shaped with a size range of 1−2 mm in diameter (Figure S1a). The size of the cells was observed by light microscopy. The cells produced ellipsoidal endospores that were positioned terminally (Figure S1b), and the cells were motile. Catalase and oxidase activity were positive. According to API 50CH tests, reactions of galactose, sorbose, rhamnose, dulcitol, α-metyl-D-glucoside, arbutin, esculin, melibiose, sucrose, trehalose, and D-turanose were positive but the other three reference strains were negative. With API 20NE, strain 03113^T^ was positive for lysine, but the other three reference strains were negative. The phenotypic properties differentiating between strain 03113^T^ and its closest phylogenetic neighbors are shown in Table 1. 

### 3.2. Analysis of Isoprenoid Quinones, Polar Lipids, and Cellular Fatty Acids

The main isoprenoid quinone of strain 03113^T^ was identified as MK-7. The polar lipids detected were diphosphatidylglycerol, phosphatidylglycerol, phosphatidylethanolamine, phosphatidylserine, three unknown aminophospholipids, and one unknown phospholipid, which was consistent with the predominant component of *B. solani* CCTCC AB 2014277^T^ [7]. The major fatty acids of strain 03113^T^ were anteiso-C_15:0_ (51.35%), iso-C_15:0_ (11.06%), and iso-C_14:0_ (7.13%), which were similar to those of the reference strains (Table 2). Iso- and anteiso- branched fatty acids of the 14-17 carbon series are typical for the genus *Bacillus* [36], which indicated that strain 03113^T^ is a member of this genus. However, the proportions of the novel strain were different from *B. solani* CCTCC AB 2014277^T^, *B. praedii* CCTCC AB 2015208^T^, and *B. dabaoshanensis* CCTCC AB 2013260^T^. For instance, the content of anteiso-C_15:0_ in strain 03113^T^ was much higher than in the reference strains, but the concentration of iso-C_15:0_ was much lower than in the related reference strains.

### 3.3. Phylogenetic Analysis of 16S rRNA

**Figure 1:**
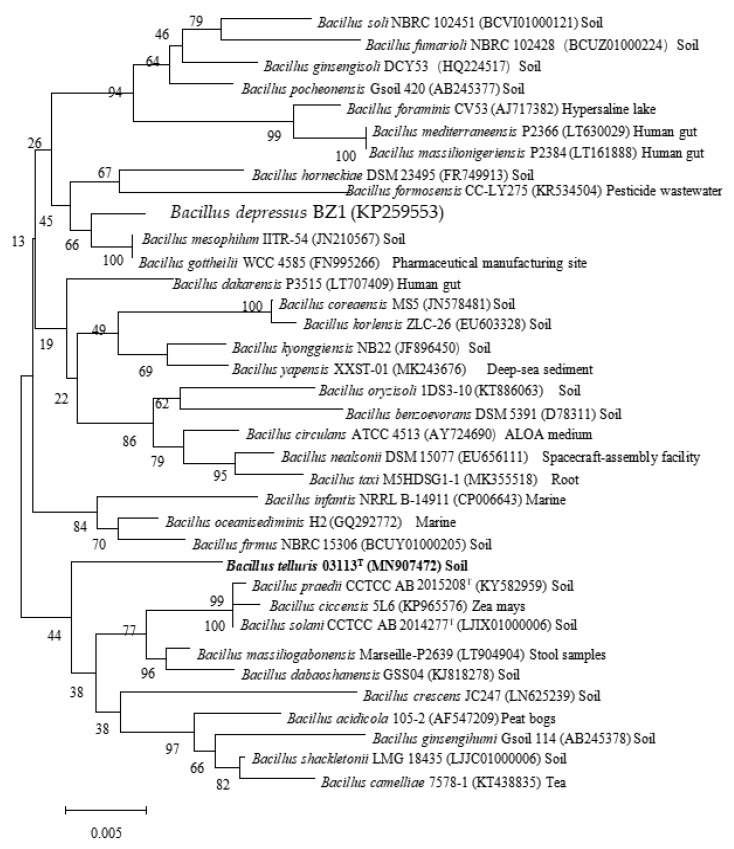
Neighbour-joining phylogenetic tree based on the 16S rRNA gene sequence of strain 03113^T^ and other closely related *Bacillus* species. The significance of each branch is indicated by a bootstrap value (%) calculated for 1000 subsets. Genbank accession numbers are given in parentheses. Bar, 0.005 substitutions per nucleotide position. Isolating source label has been annotated in the back.

The complete 16S rRNA gene sequence (1347 bp) was discovered from the draft genome of the novel strain. Pairwise comparisons showed that strain 03113^T^ was related most closely to *B. solani* CCTCC AB 2014277^T^ (98.14% similarity), followed by *B. praedii* CCTCC AB 2015208^T^ (98.07%), and *B. dabaoshanensis* CCTCC AB 2013260^T^ (98.0%). Phylogenetic trees were reconstructed using the maximum-likelihood, neighbour-joining, and minimum-evolution methods. All three treeing methods yielded a similar phylogeny. Strain 03113^T^ was located within the genus *Bacillus* and had a separated clade based on the phylogenetic trees of 16S rRNA genes (Figure 1, Figure S2, Figure S3), indicating that 03113^T^ was a novel species of genus *Bacillus*.

### 3.4. Whole-Genome Analysis

**Figure 2:**
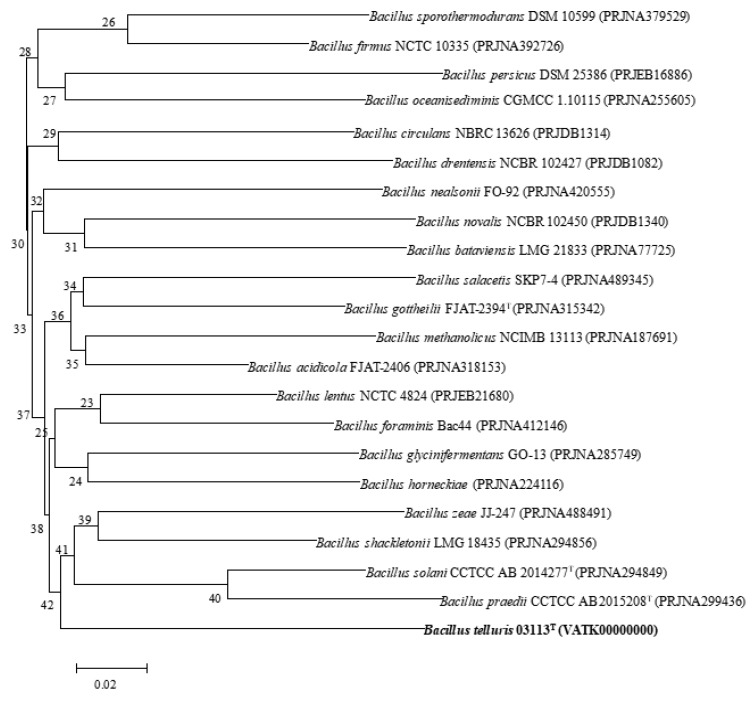
Phylogenomic tree generated with Genome-to-Genome Distance Calculator (GGDC), showing the phylogenomic position of strain 03113^T^ and the type strains of related species of *Bacillus*. The numbers at the nodes indicate the gene support index. Bar, 0.02 substitutions per position.

A total of 5,033,596 reads were obtained from draft genome sequencing of strain 03113^T^, which yielded a genome of 4,856,532 reads in length. *N50* value was 190,698 bp, and the largest contig was 198,446 bp. The genome was predicted to contain a total of 4288 genes, which included 4241 protein-coding genes, 2 rRNA genes, and 45 tRNA genes. The genomic DNA G+C content of strain 03113^T^ was 36.08 mol%. The phylogenomic tree based on the GGDC web also revealed the distinct phylogeny of strain 03113^T^ and its close relationship with *B. solani* CCTCC AB 2014277^T^, *B. praedii* CCTCC AB 2015208^T^, and *B. dabaoshanensis* CCTCC AB 2013260^T^ (Figure 2). ANIb and ANIm values of strain 03113^T^ with the type strain of the most closely related species, *B. solani* CCTCC AB 2014277^T^, were 70.5% and 85.9%, respectively. All ANI values were much lower than the 96.0% cut-off value that was proposed previously for the genus *Bacillus* [38,39]. The DDH value of strain 03113^T^ and *B. solani* CCTCC AB 2014277^T^ was 22.6%, which was much lower than 70%. The ANI and DDH between strain 03113^T^ and the other reference species *B. praedii* CCTCC AB 2015208^T^ were 70.5% and 20.9%, respectively. This genome sequence, which was deposited in the GenBank/EMBL/DDBJ database under accession number VATK00000000, was used for further analysis. Thus, complete genome analysis combined with 16S rRNA phylogenetic, physiological, and biochemical properties all supported the conclusion that strain 03113^T^ should be considered a novel species in the genus *Bacillus*. 

### 3.5. Phylogenomic Comparative Analysis of Bacillus species

Based on the above database, we conducted a preliminary analysis of the pan-genome, which showed that 840 shared orthologous coding sequences were clustered into the core genome of *Bacillus*, 32,926 were represented in the accessory genome, and 22,024 were identified as strain-unique genes (Figure S4a). Therefore, a highly reliable mathematical extrapolation of the pan and core genome was constructed (Figure S4b). The total genes increase in the pan-genome of *Bacillus* with the rise in the analyzed genome number, suggesting that the pan-genome was open. The previous reports showed that the genes’ number of core genomes was highly conserved, while many strain-unique genomes and accessory genomes are thought to contribute to species diversity [40], which indicated that species in the genus *Bacillus* were also multifarious. A phylogenetic tree reconstructed based on the concatenated alignment of these 840 core orthologous proteins (Figure S4c) showed that strain 03113^T^ clustered closely with known species, indicating that it was a member of the genus *Bacillus*. This is consistent with the previous results.

### 3.6. Plant Growth-Promoting Characteristics of Isolates

The qualitative determination indicated that strain 03113^T^ secreted auxin IAA, and the colour reaction was pink, at a concentration of 175.94 μg/mL (Figure S5). Strain 03113^T^ did not generate a color ring on the CAS flat plate, and no clear zone was observed around each of the colonies of strain 03113^T^ on inorganic or organic phosphate media, which indicated that it did not produce siderophores or dissolve phosphate. 

Strain 03113^T^ carried the nitrogenase iron protein (*nifH*) gene, based on the genome annotation, which is a key enzyme for fixing nitrogen in bacteria. The *nifH* gene of 03113^T^ had a very low similarity with published sequences based on Blast in Genbank (https://blast.ncbi.nlm.nih.gov/Blast.cgi). It was mostly related to *B. alkalidiazotrophicus* MS6 and *B. arsenniciselenatis* E1H with a similarity of 80%, which indicated that strain 03113^T^ has the potential to fix nitrogen. 

## 4. Conclusions

From the phenotypic and chemotaxonomic properties of strain 03113^T^, 16S rRNA gene sequence comparisons, and DNA–DNA hybridization, we concluded that strain 03113^T^ (=ACCC 03113^T^=JCM 33017^T^) was distinguished from the known species in the genus *Bacillus*. Based on the present polyphasic analysis, strain 03113^T^ is considered to represent a novel species within the genus *Bacillus*, for which we propose the name *Bacillus telluris* sp. nov. The description of *Bacillus telluris* sp. nov. is summarized in Appendix A.

## Figures and Tables

**Table 1:** Differential phenotypic characteristics of strain 03113^T^ and closely related strains in the genus *Bacillus*.

Characteristic	1	2	3	4
Optimal growth conditions				
Temperature for growth (°C)	37	30–37	35	30
pH for growth	8.0	7.0	9.0	9.0
NaCl concentration for growth (%, *w/v*)	5	1	0	4
The Acid produced from (API 50CH)				
L-arabinose	+	+	+	−
Esculin	+	+	−	−
API 20NE				
β-galactosidase	+	+	−	−
Lysine	+	−	−	−
Lohn gelatin	+	+	+	−
Utilization (Biolog GEN III)				
dextrin	+	+	−	+
d-maltos, d-trehalose, sucrose, d-turanose, d-raffinose	+	−	−	−
*N*-acetyl-d-glucosamine	−	+	+	−
*N*-acetyl-β-d-mannosamine, acetic acid	−	+	−	−
Stachyose, d-mannose	w	−	−	−
*N*-acetyl-d-galactosamine	−	w	−	−
α-d-glucose	+	−	w	−
d-fructose	+	+	w	−
Inosine, d-serine, glycerol, d-glucose-6-PO_4_, d-fructose-6-PO_4_, nalidixic acid, lithium chloride, aztreonam, lincomycin	−	+	+	+
Troleandomycin	−	−	+	−
L-aspartic acid, L-glutamic acid, L-histidine, L-pyroglutamic acid, L-serine, L-lactic acid, sodium butyrate	-	+	+	−

Strains: 1, 03113^T^; 2, *B. dabaoshanensis* CCTCC AB 2013260^T^; 3, *B. solani* CCTCC AB 2014277^T^; 4, *B. praedii* CCTCC AB 2015208^T^. All strains were negative for sodium thiosulfate, tryptophan, d-cellobiose, gentiobiose, α-d-lactose, d-melibiose, β-methyl-d-glucoside, d-salicin, *N*-acetyl neuraminic acid, d-galactose, 3-methyl glucose, L-fucose, L-rhamnose, fusidic acid, d-sorbitol, d-mannitol, myo-inositol, d-aspartic acid, minocycline, pectin, d-galacturonic acid, glucuronamide, mucic acid, quinic acid, p-hydroxy-phenylacetic acid, citric acid, α-keto-glutaric acid, d-malic acid, γ-amino-butryric acid, α-hydroxy-butyric acid, β-hydroxy-D, L-butyric acid, α-keto-butyric acid, propionic acid, and formic acid. All strains were positive for sodium lactate and potassium tellurite. All data were from the present study. +, positive; w, weakly positive; −, negative.

**Table 2:** The cellular fatty acid content of strain 03113^T^ and representative strains of closely related species of the genus *Bacillus*. Strains: 1, 03113^T^; 2, *B. dabaoshanensis* CCTCC AB 2013260^T^; 3, *B. solani* CCTCC AB 2014277^T^; 4, *B. praedii* CCTCC AB 2015208^T^. All data were obtained in this study. Partial values lower than 1% are not shown in the table. ND, Not detected.

Fatty acid	1	2^a^	3	4
C_14:0_	1.96	1.1	1.77	1.68
C_16:0_	7.50	2.60	1.98	2.20
iso-C_14:0_	7.13	ND	6.61	5.13
iso-C_15:0_	11.06	42.9	45.43	54.12
iso-C_16:0_	8.73	6.7	6.07	5.61
anteiso-C_15:0_	51.35	24.1	27.16	20.15
anteiso-C_17:0_	6.71	6.2	3.88	3.13
C_16:1_*ω*7c alcohol	ND	ND	2.56	2.36
C_16:1_*ω*w11c	ND	ND	1.21	1.12
Summed Feature 3 *	<1	2.5	ND	ND
Summed Feature 8 ^†^	<1	1.5	ND	ND

* Summed feature 3 comprises C_16: 1_*ω*6c and/or C_16: 1_*ω*7c. † Summed feature 8 comprises C_18: 1_*ω*6c and/or C_18: 1_*ω*7c.^a^ Data were obtained from: Cui et al. [37].

## Supplementary Materials

The following are available online at https://www.mdpi.com/2076-2607/8/5/702/s1, Figure S1: (**a**) The morphology and Gram-staining of strain 03113^T^ of the genus *Bacillus*. (**b**) The morphology and Spore-staining of the strain 03113^T^ in the genus *Bacillus*. Figure S2: Maximum-Likelihood phylogenetic tree based on the 16S rRNA gene sequence of strain 03113^T^ and other closely related *Bacillus* species. The significance of each branch is indicated by a bootstrap value (%) calculated for 1000 subsets. Genbank accession numbers are given in parentheses. Bar, 0.005 substitutions per nucleotide position. Figure S3: Minimum-Evolution phylogenetic tree based on the 16S rRNA gene sequence of strains 03113^T^ and other closely related *Bacillus* species. The significance of each branch is indicated by a bootstrap value (%) calculated for 1000 subsets. Genbank accession numbers are given in parentheses. Bar, 0.005 substitutions per nucleotide position. Figure S4: The Pan-genome analysis and the genome phylogenetic tree of strains belonging to the *Bacillus* genus. (**a**) Petal diagram of the pan-genome. Each strain is represented by a colored oval. The center is the number of orthologous coding sequences shared by all strains. Numbers in nonoverlapping portions of each oval show the numbers of CDSs unique to each strain. (**b**) Mathematical modeling of the pan-genome and core genome of *Bacillus*. (**c**) Tree constructed according to the maximum-likelihood method based on 840 core orthologous proteins of strain 03113^T^ and closely related species of the genus *Bacillus*. Bootstrap values are expressed as percentages of 1000 replications, and those over 70% are shown at branch points. Bar, 0.05 substitutions per nucleotide position. Figure S5: Colour reaction of strain 03113^T^ compared to CK (CK: Salkowski solution mixed with IAA). Figure S6: Polar lipids of strain 03113^T^ after two-dimensional TLC and detection with (I) molybdophosphoric acid spray reagent and heating at 150 °C for 10 min, (II) molybdenum blue spray reagent, and (III) ninhydrin spray reagent and heating at 100 °C for 10 min. No spots were detected by α-naphthhol spray reagent and heating at 100 °C for 5 min. Chloroform/methanol/water (65:25:4, by vol.) was used in the first direction. (1), followed by chloroform/acetic acid/methanol/water (80:15:12:4, by vol.) in the second direction. (2), DPG, diphosphatidylglycerol; PG, phosphatidylglycerol; PE, phosphatidylethanolamine; PSer, phosphatidylserine; APL1-3, unknown aminophospholipid; PL1, unknown phospholipids.

## Appendix A

Description of *Bacillus telluris* sp. nov.

*Bacillus telluris* (tel. lu′ris. L. gen. n. telluris from soil, the origin of the strain).

Cells are Gram-stain-positive, endospore-forming rods, motile, about 1−2 mm in diameter. Colonies are circular and smooth on TSA at 30 °C. Ellipsoidal endospores were observed at the terminal position. Growth occurred at 4 °C −50 °C (optimum 37 °C), at pH 3.0−12.0 (optimum pH 8.0), and with 0–10% (*w/v*) NaCl (optimum 5%). Catalase and oxidase activity were all positive. It produced the biological characteristics of IAA. Reactions of L-arabinose, ribose, D-xylose, galactose, sorbose, rhamnose, dulcitol, α-metyl-D-glucoside, amygdalin, arbutin, esculin, maltose, melibiose, sucrose, trehalose, D-turanose, and gluconate were positive. Positive reactions for β-galactosidase (ONPG), arginine, lysine, sodium citrate, urease, pyruvate, and kohn gelatin, but negative reactions for H_2_S production, indole production, ornithine, tryptophan, glucose, mannose, inositol, sorbitol, rhamnose, sucrose, melibiose, amygdalin, and arabinose. Dextrin, D-maltose, D-maltose, D-trehalose, sucrose, D-turanose, D-raffinose, α-D-glucose, stachyose, D-mannose, D-fructose, and sodium lactate were assimilated, but gentiobiose, D-melibiose, D-fructose, D-serine, myo-inositol, gelatin, and D-glucuronic acid were not. The main cellular fatty acids were anteiso-C_15:0_, iso-C_15:0_, and iso-C_14:0_. Major polar lipids were diphosphatidylglycerol, phosphatidylglycerol, phosphatidylethanolamine, and phosphatidylserine (Figure S6). The main isoprenoid quinone was MK-7.

The strain, 03113^T^ (=ACCC 03113^T^=JCM 33017^T^), was isolated from greenhouse soil collected in Wangsiying, Chaoyang District, Beijing, China. The DNA G+C content of the genome of the strain was 36.08 mol%.

The GenBank/EMBL/DDBJ accession number for the 16S rRNA sequence of *Bacillus telluris* 03113^T^ is MN907472, and the complete genome is deposited under the accession number VATK00000000.
