# Supplementary material for: Bacillus telluris sp. nov. Isolated from Greenhouse Soil in Beijing, China
Source: Microorganisms. 2020 May 10;8(5):702. doi: 10.3390/microorganisms8050702 (PMC7285132; doi:10.3390/microorganisms8050702)
Supplement: Supplementary file 1 [file microorganisms-08-00702-s001.pdf]

Bacillus telluris sp. nov. Isolated from Greenhouse Soil in Beijing, China

He-Bao Guo,[1] Shan-Wen He,[1] Xing Wang,[1] Kyu -Kyu Thin,[1] Hai-Lei Wei[1]* and Xiao-Xia Zhang[1]*

[1] Key Laboratory of Microbial Resources Collection and Preservation, Ministry of Agriculture and Rural Affairs, Institute of Agricultural Resources and Regional Planning, Chinese Academy of Agricultural Sciences, Beijing 100081, PR China.

* Correspondence: weihailei@caas.cn; zhangxiaoxia@caas.cn

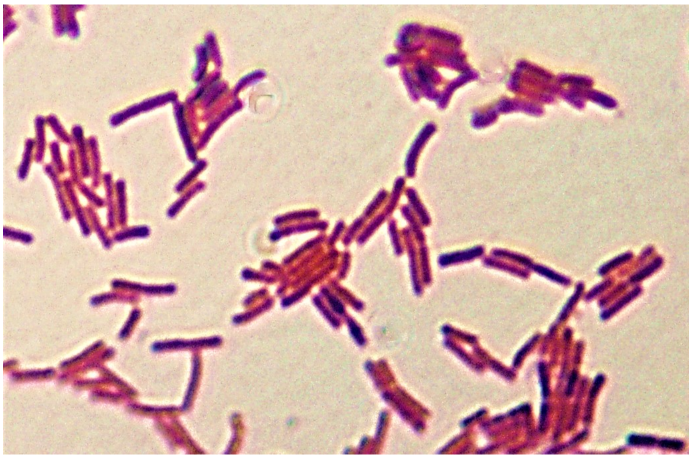

Figure S1. (a) The morphology and Gram-staining of strain 03113^T of the genus *Bacillus*.

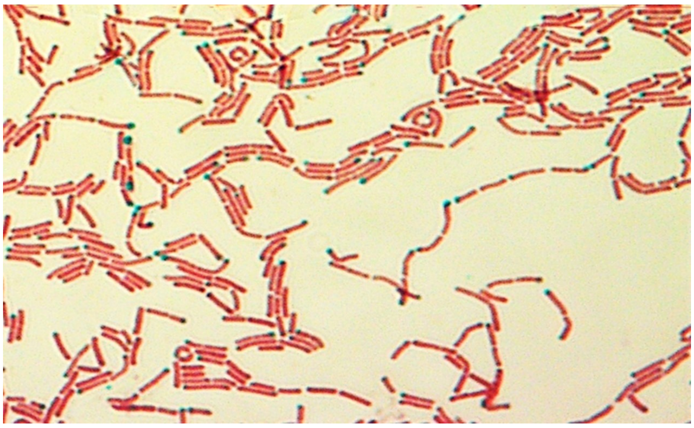

Figure S1. (b) The morphology and Spore-staining of the strain 03113^T in the genus *Bacillus*.

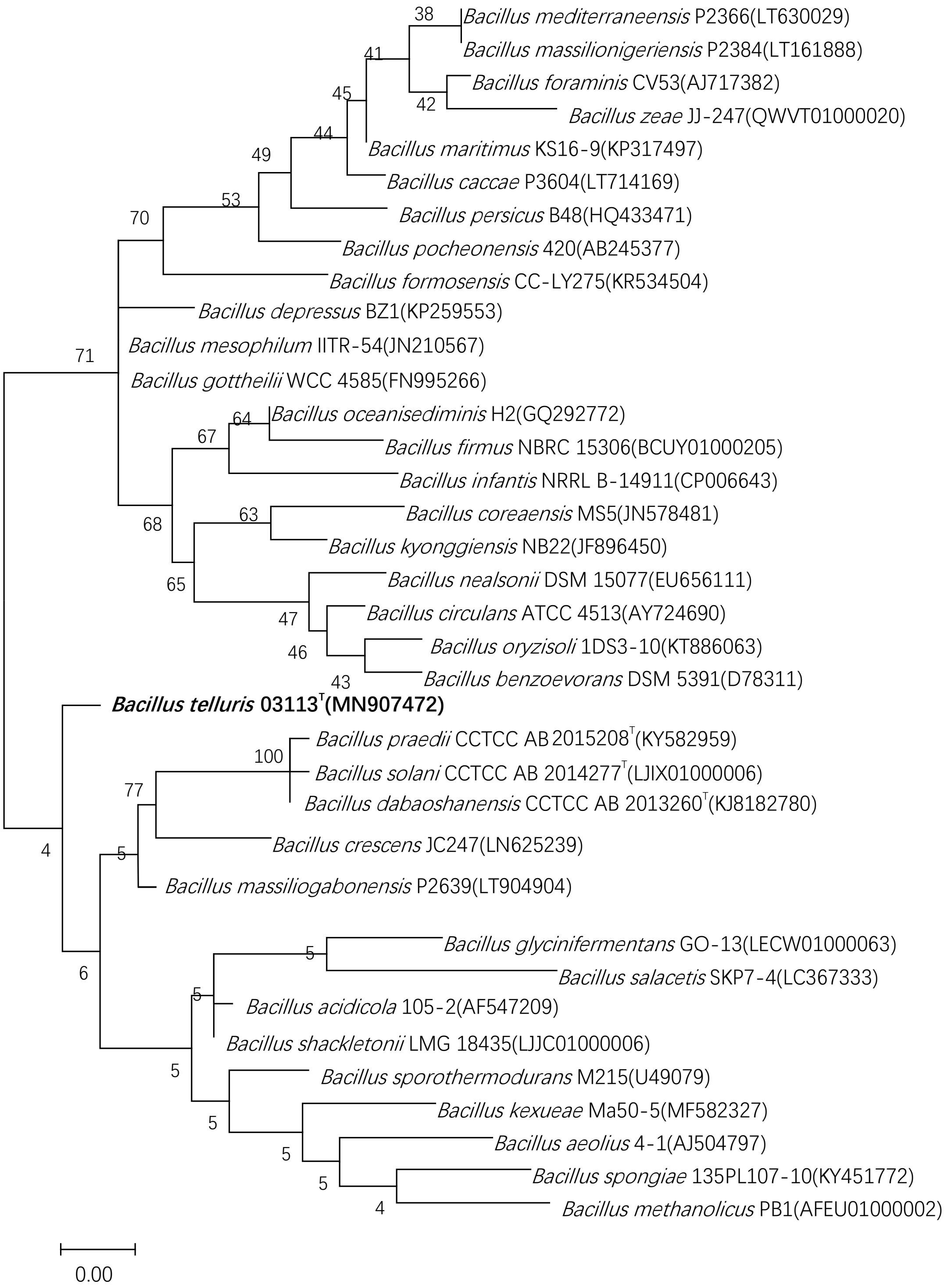

Figure S2. Maximum-Likelihood phylogenetic tree based on the 16S rRNA gene sequence of strains 03113^T and other closely related *Bacillus* species. The signi□cance of each branch is indicated by a bootstrap value (%) calculated for 1000 subsets. Genbank accession numbers are given in parentheses. Bar, 0.005 substitutions per nucleotide position.

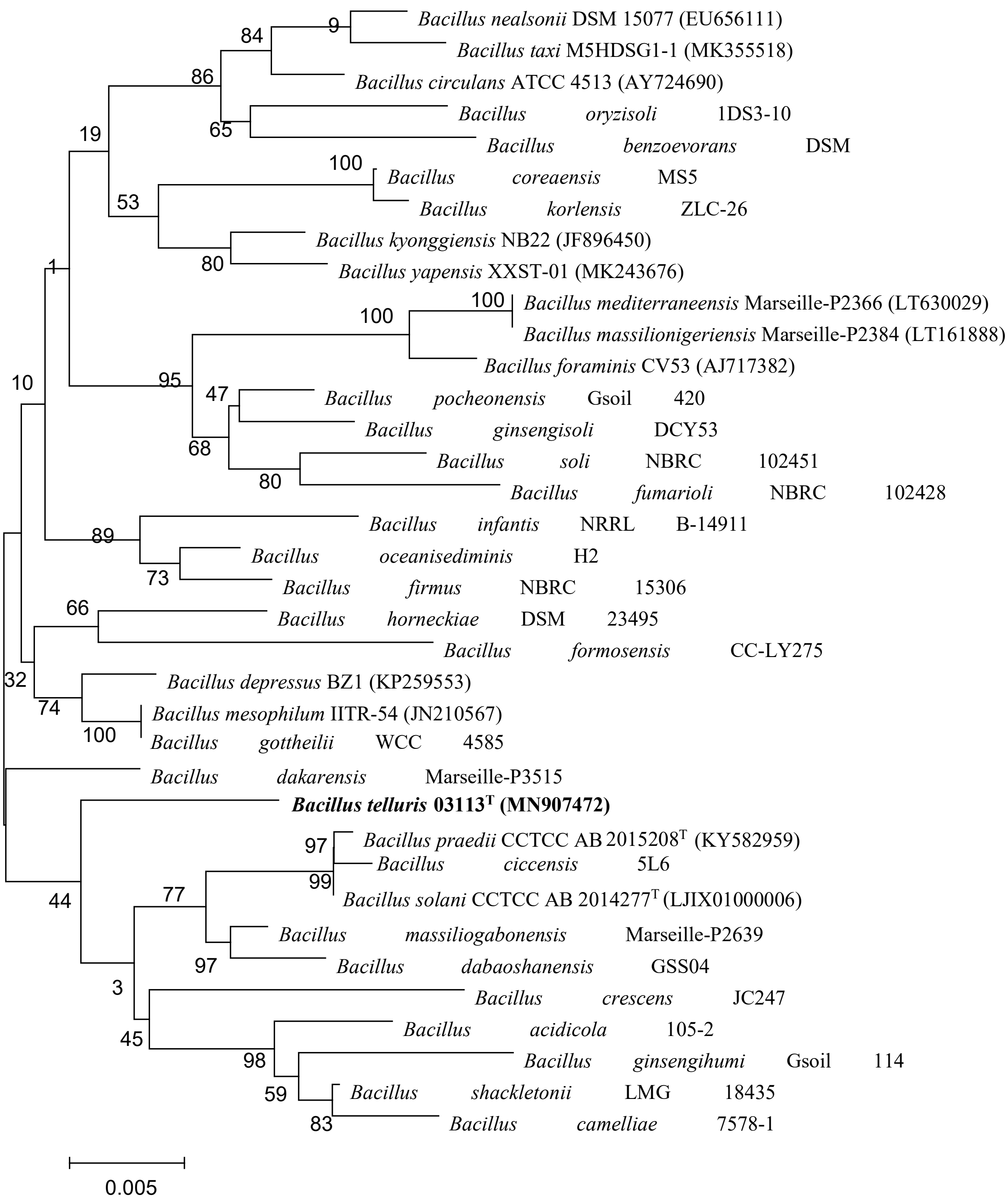

Figure S3. Minimum-Evolution phylogenetic tree based on the 16S rRNA gene sequence of strains 03113^{T} and other closely related *Bacillus* species. The significance of each branch is indicated by a bootstrap value (%) calculated for 1000 subsets. Genbank accession numbers are given in parentheses. Bar, 0.005 substitutions per nucleotide position.

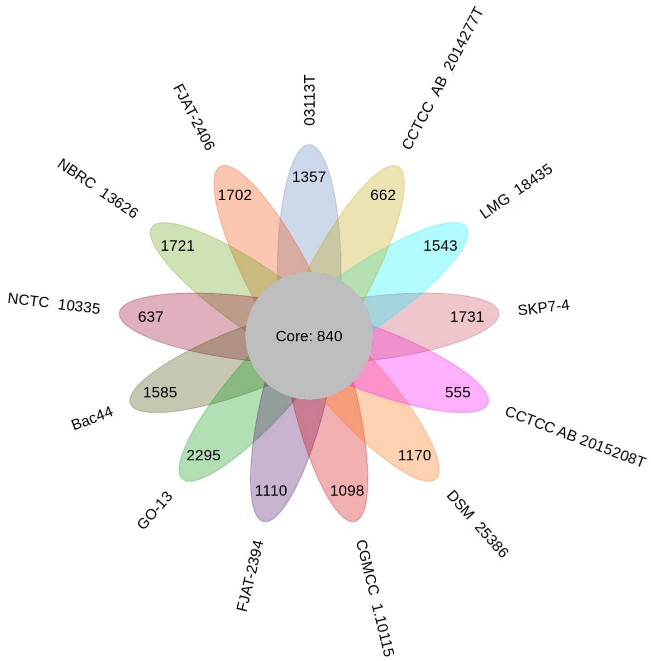

(a)

Figure S4. The Pan-genome analysis and the genome phylogenetic tree of strains belonging to the *Bacillus* genus. (a) Petal diagram of the pan-genome. Each strain is represented by a colored oval. The center is the number of orthologous coding sequences shared by all strains. Numbers in nonoverlapping portions of each oval show the numbers of CDSs unique to each strain.

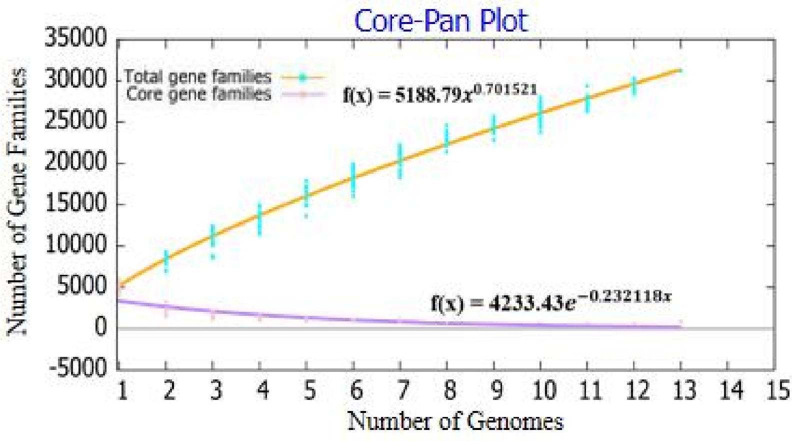

(b)

Figure S4. (b) Mathematical modeling of the pan genome and core genome of *Bacillus*.

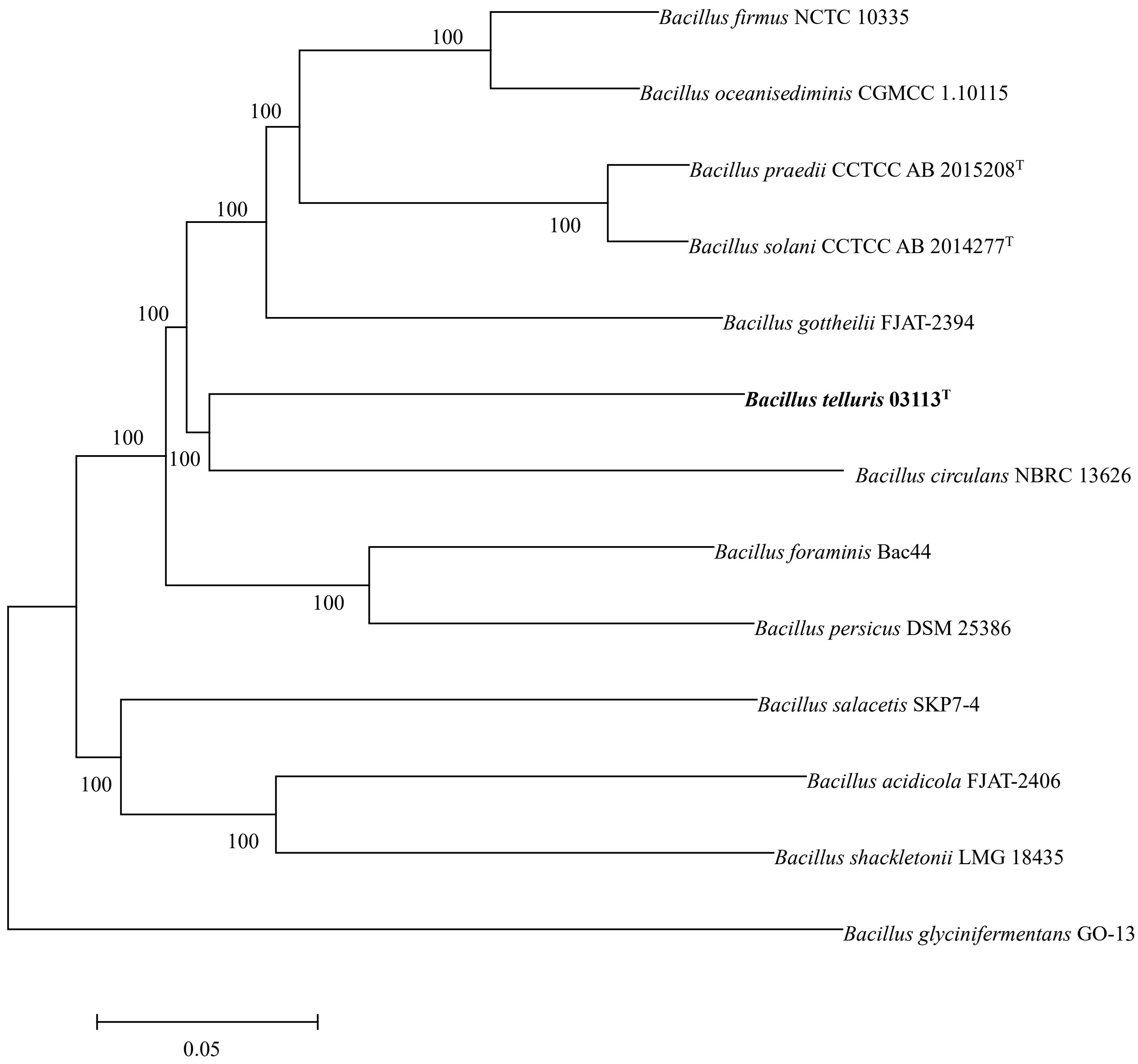

Figure S4. (c) Tree constructed according to the maximum-likelihood method based on 840 core orthologous proteins of strain 03113^T and closely related species of genus *Bacillus*. Bootstrap values are expressed as percentages of 1000 replications, and those over 70% are shown at branch points. Bar, 0.05 substitutions per nucleotide position.

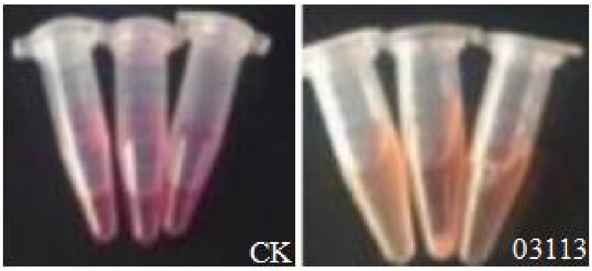

Figure S5. Colour reaction of strain 03113^T compared to CK (CK: Salkowski colorimetric mixed with IAA)

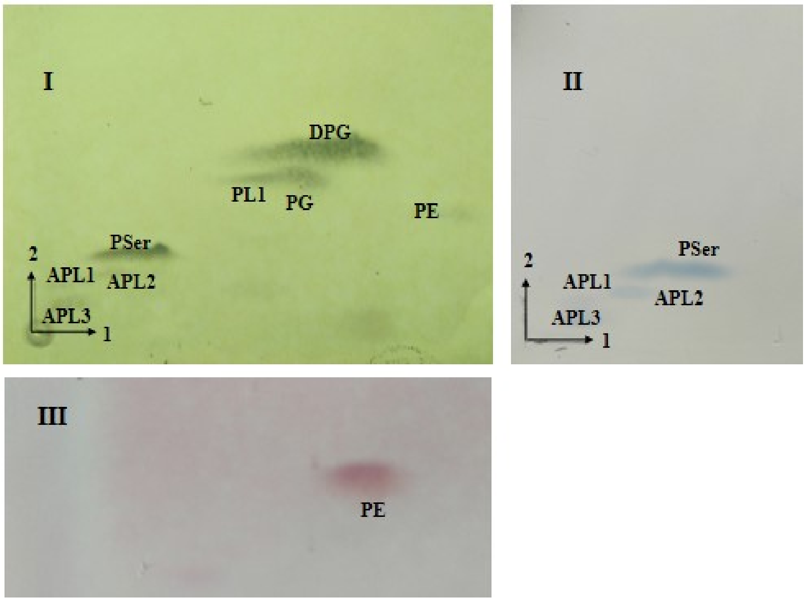

Figure S6. Polar lipids of strain 03113^T after two-dimensional TLC and detection with (I) molybdophosphoric acid spray reagent and heating at 150°C for 10 min, (II) molybdenum blue spray reagent, and (III) ninhydrin spray reagent and heating at 100°C for 10 min. No spots were detected by α-naphthhol spray reagent and heating at 100°C for 5 min. Chloroform/methanol/water (65:25:4, by vol.) was used in the first direction. (1), followed by chloroform/acetic acid/methanol/water (80:15:12:4, by vol.) in the second direction. (2), DPG, diphosphatidylglycerol; PG, phosphatidylglycerol; PE, phosphatidylethanolamine; PSer, phosphatidylserine; APL1-3, unknown aminophospholipid; PL1, unknown phospholipids.
